# Simultaneous Measurement of Thirteen Steroid Hormones in Women with Polycystic Ovary Syndrome and Control Women Using Liquid Chromatography-Tandem Mass Spectrometry

**DOI:** 10.1371/journal.pone.0093805

**Published:** 2014-04-08

**Authors:** Candace C. Keefe, Mildred M. Goldman, Ke Zhang, Nigel Clarke, Richard E. Reitz, Corrine K. Welt

**Affiliations:** 1 Reproductive Endocrine Unit, Massachusetts General Hospital, Boston, Massachussetts, United States of America; 2 Steroids Department, Quest Diagnostics Nichols Institute, San Juan Capistrano, California, United States of America; University of North Carolina at Chapel Hill, United States of America

## Abstract

**Background:**

The measurement of adrenal and ovarian androgens in women with PCOS has been difficult based on poor specificity and sensitivity of assays in the female range.

**Methods:**

Women with PCOS (NIH criteria; n = 52) and control subjects with 25–35 day menstrual cycles, no evidence of hyperandrogenism and matched for BMI (n = 42) underwent morning blood sampling. Liquid chromatography-tandem mass spectrometry (LC-MS/MS) was used to simultaneously measure 13 steroids from a single blood sample to measure adrenal and ovarian steroids. Androgen and progesterone results were compared in the same samples using RIA.

**Results:**

Testosterone, androstenedione, progesterone and 17OH progesterone levels were higher when measured using RIA compared to LC-MS/MS, although the testosterone RIA demonstrated the best agreement with the LC-MS/MS using a Bland-Altman analysis. Results using LC-MS/MS demonstrated that the concentration of androgens and their precursors were higher in women with PCOS than controls [median (2.5, 97.5th %ile); 1607 (638, 3085) vs. 1143 (511, 4784) ng/dL; p = 0.03]. Women with PCOS had higher testosterone [49 (16, 125) vs. 24 (10, 59) ng/dL], androstenedione [203 (98, 476) vs. 106 (69, 223) ng/dL] and 17OH progesterone levels [80 (17, 176) vs. 44 (17, 142) ng/dL] compared to controls (all P<0.02), but no differences in serum concentrations of the adrenal steroids DHEAS, cortisol, corticosterone and their 11 deoxy precursors. Women with PCOS also had an increase in the product:precursor ratio for 3β-hydroxysteroid dehydrogenase [22% (6, 92) vs. 20% (4, 43); p = 0.009].

**Conclusion:**

LC-MS/MS was superior to RIA in measuring androstenedione, progesterone and 17OH progesterone levels, while testosterone measurements were better matched in the two assays. Androgen levels were higher in women with PCOS in the absence of a difference in adrenal-predominant steroids. These data support previous findings that the ovary is an important source for the androgen excess in women with PCOS.

## Introduction

Hyperandrogenism is a cardinal feature of polycystic ovary syndrome (PCOS) [1]. Both ovarian and adrenal androgen sources contribute to serum androgen levels. The increased androgen production of the theca cells in the ovary has been well established [2], [3]. In addition, there is some evidence for increased adrenal steroidogenesis compared to controls [4]. However, our own data do not demonstrate elevation of DHEAS, an adrenal specific androgen, in women with PCOS [5]. Thus, the importance of the adrenal contribution to androgen secretion remains to be determined [6].

Despite the importance of accurate androgen measurements in PCOS, most of the clinically available androgen immunoassays have problems with sensitivity, specificity and accuracy. The difficulties are exemplified by direct immunoassays and extraction-only radioimmunoassays (RIAs) for testosterone, which perform well for testosterone levels in the range of healthy, young men but do not have sufficient sensitivity and accuracy at the concentrations found in women and children [7]. However, some investigators have demonstrated that RIA can perform as well as LC-MS/MS [8], [9]. In addition to improved accuracy and sensitivity for steroid measurements, liquid chromatography-tandem mass spectrometry (LC-MS/MS) can separate compounds and can therefore be used to measure multiple steroids from one sample [10], [11].

The current study examines a new LC-MS/MS for the simultaneous determination of 13 steroids. The testosterone, androstenedione, progesterone and 17-hydroxyprogesterone measurements were compared to those determined using radioimmunoassays and immunoassays. The simultaneous measurement of 13 steroid hormones using LC-MS/MS was then used to evaluate ovarian and adrenal steroids in women with PCOS and to estimate the relative steroid enzyme activity in women with PCOS compared to controls [12].

## Methods

### Subjects

Women with PCOS were selected at random from a large group of study participants (n = 52) [5]. They had 1) hyperandrogenism manifested by acne (presence of acne lesions) or hirsutism (Ferriman-Gallwey score>9; [13]) and 2) 8 or fewer menses per year, fulfilling the NIH criteria [14], and a BMI between 19–57 kg/m^2^. PCOS subjects were at least 10 days after a vaginal bleed to avoid the known suppression of LH and androgens after progesterone exposure [13].

Healthy control subjects (n = 42), aged 18–35 years, had regular menstrual cycles (25–35 days), and normal testosterone levels (<63 ng/dL) [5]. They were chosen from a large group of study participants to match the average BMI of the PCOS participants (20–41 kg/m^2^).

All subjects were recruited between 2004 and 2010, arrived between 8 and 9 a.m. in the follicular phase and underwent a fasting blood sample at time 0 and 10 minutes through the same catheter. The 10 minute sample was used for LC-MS/MS to avoid capturing hormonal changes related to stress.

An additional control group of healthy women was recruited in 2010 to provide normative data. The subjects were aged 18–35 years, with a body mass index (BMI) between 18–30 kg/m^2^ (n = 32). Subjects reported for daily blood draws, at the same time each day, across one menstrual cycle. Each subject was allowed to miss up to 4 samples in the cycle. Up to three pelvic ultrasounds were performed between days 7 and 19 to ensure the emergence of a dominant follicle and ovulation.

All subjects had normal thyroid function and prolactin levels. They were on no regular medication, and did not take hormonal contraception for at least 3 months before study enrollment. The studies were approved by the Partners Human Research Committee and all subjects gave written informed consent.

### Assays

RIA and immunoassays were performed on fresh serum. Samples underwent one freeze/thaw cycle before LC-MS/MS. Our laboratory has demonstrated that testosterone levels are stable (less than ±15%, which is comparable to typical reagent lot based changes) on serum samples stored up to 14 years (Dr. Patrick Sluss, personal communication; Figure S1 in File S1). The Quest Diagnostics Nichols Institute LC-MS/MS multiplex assay was used and allows for simultaneous analysis of up to 13 steroids (San Juan Capistrano, CA). Both the extraction columns (Cyclone-P 2.0×5.0 mm) and analytical columns (Hypersil Gold C8, 5 μm 100×2.1 mm) were purchased from Thermo Scientific.

#### Reagents

11-deoxycortisol, corticosterone, cortisone and deoxycorticosterone were purchased from Sigma-Aldrich Inc. (St. Louis, MO). 17-hydroxyprogesterone, 17-hydroxypregnenolone, androstenedione, DHEA, pregnenolone and testosterone were purchased from Steraloids, Inc. (Newport, RI). 18-hydroxycorticosterone and cortisone-[2,2,4,6,6,9,12,12-H] were purchased from Isosciences (King of Prussia, PA). Cortisol and progesterone were purchased from U.S. Pharmacopeia (Rockville, MD).

Three internal standards were included for 17-hydroxyprogesterone, cortisone and pregnenolone. *17α-hydroxyprogesterone-d_8_* (4-Pregnen-17α-ol-3.20-dione-2,2,4,6,6,21,21,21-d_8_) and *pregnenolone-2,2,3,4,4,6-d_6–_* were purchased from CDN Isotopes (Quebec, Canada).

Steroid-free, double charcoal-stripped serum was purchased from Golden West Biologicals (Temecula, CA) and was tested for detectable steroids before use. All other chemicals were reagent grade or better and were purchased from commercial sources.

Estradiol was measured in a separately validated LC-MS/MS commercially available assay [11]. Briefly, extraction was accomplished using a 1.0×50 mm, 50 μm Cyclone P column (ThermoFisher, San Jose, CA), and separation was accomplished by a 150×2.0 mm, 4 μm Synergi Polar-RP column (Phenomenex, Torrance, CA). Analysis was performed on a TSQ Quantum Ultra (Thermo Fisher; San Jose, CA) triple quadrupole tandem mass spectrometer using a deuterated internal standard. Serum LH and FSH were measured using a 2-site monoclonal non-isotopic system (Axsym, Abbott Laboratories, Abbott Park, IL) [15]. LH and FSH levels are expressed in IU per liter as equivalents of the Second International Reference Preparation 71/223 of human menopausal gonadotropins.

#### Comprehensive steroid panel by high turbulence liquid chromatography-MS/MS

The sample was prepared by acidifying 100 μL of serum with 20% formic acid containing the three internal standards in order to break up the ionic interaction from the carrier protein and release the analytes without precipitating. After vigorous mixing, the samples were incubated at room temperature for 15 to 20 minutes prior to being placed in the refrigerated autosampler for injection.

The Aria TLX System (Thermo Electron - Franklin, MA) injected the prepared sample which was then loaded in a high aqueous mobile phase solvent onto an extraction column (Cyclone-P 2.0×5.0 mm) at a high flow rate (5 mL/min). This created turbulence inside the column, which allowed the steroids to bind to the large particles of the extraction column, while protein and other debris freely flowed through and were discarded. Following the loading step, the flow was reversed and the sample was eluted off the extraction column and transferred to a reverse-phase C8 analytical column. A binary high performance liquid chromatography gradient was applied to the column resulting in the separation of the 13 steroids and 3 internal standards from each other and their metabolites.

The steroids were quantified using a TSQ Quantum Ultra (Thermo Fisher; San Jose, CA) triple quadrupole tandem mass spectrometer. The tandem mass spectrometer permits the isolation of the parent compound within±0.5 m/z within the first quadrupole (Q1). In the second quadrupole (Q2), the parent ions collide with an inert gas (argon) to generate daughter ions which are selected in the third quadrupole (Q3). Inclusion of deuterated steroids such as cortisone, 17-hydroxyprogesterone and 17-hydroxypregnenolone as internal standards enabled absolute quantification of the steroids by correcting for procedural losses or ion suppression caused by matrix effects in the atmospheric pressure chemical ionization (APCI) process. The intra-assay coefficients of variation (CV) for 10 replicates of a sample ranged from 2.7 to 13.2% at three concentration levels (Table S1 in File S1), The inter-assay CVs, determined using 10 replicates from each of 3 quality control pools and run over 5 days (n = 50), ranged from 4.6 to 13.1% (Table S2 in File S1). All of the assays demonstrated a correlation coefficient greater than 0.99 for a calibration range of 25 to 10,000 ng/dL, or 0.05 to 10 μg/dL for cortisol, 0.025 to 10 μg/dL for cortisone and 0.25 to 100 ng/dL for progesterone (Table S3 in File S1). Recovery was within ±10% expected for all steroids.

### Statistics

Linear regression and Bland-Altman plots were used to compare LC-MS/MS results for each individual with immunoassay results for progesterone and RIA results for testosterone, 17-hydroxyprogesterone and androstenedione. The difference between assay results for individuals from the RIA and LC-MS/MS was calculated as a percentage of the mean level measured by the two assays for each hormone. The percent difference between assay results was also compared in women with PCOS and controls using a *t* test.

Comparisons between steroid levels measured using LC-MS/MS in women with PCOS and controls studied on a single day were performed using a Mann-Whitney Rank Sum test and logistic regression with BMI and age as covariates (Table 1). Steroid product-to-precursor ratios were used to estimate enzyme activity for androgen and cortisol synthesis and were compared between women with PCOS and controls. A p value of <0.05 was considered significant.

**Table 1:** Age, BMI and steroid concentrations in women with PCOS and controls in the follicular phase.

	PCOS (n = 52)	Controls (n = 42)	P Value	P value corr^1^
**Age (yrs)**	25 (18, 38)^2^	30 (19, 40)	0.1	NA^3^
**BMI (kg/m** 2 **)**	26.6 (20.2, 48.4)	27.8 (21.1, 33.3)	0.4	0.2
**Testosterone (ng/dL)**	49.2 (16.2, 125.3)	24.2 (10.0, 58.8)	<0.001	<0.001
**Androstenedione (ng/dL)**	202.9 (98.2, 476.2)	106.2 (68.9, 223.2)	<0.001	<0.001
**17OH Progesterone (ng/mL)**	79.6 (17.0, 175.5)	44.3 (17.0, 142.5)	0.006	0.02
**17OH Pregnenolone (ng/mL)**	477.8 (44.4, 2219.1)	333.0 (33.0, 3550.3)	0.5	0.6
**Pregnenolone (ng/dL)**	35.0 (35.0, 615.2)	35.0 (35.0, 168.9)	0.4	0.8
**Progesterone (ng/dL)**	0.12 (0.08, 0.29)	0.13 (0.08, 0.88)	0.4	0.06
**Deoxycorticosterone (ng/dL)**	16.0 (16.0, 48.4)	16.0 (16.0, 69.5)	0.8	0.8
**Corticosterone (ng/dL)**	151.2 (32.1, 605.5)	201.8 (64.9, 747.8)	0.2	0.2
**11 Deoxycortisol (ng/dL)**	15.0 (15.0, 57.4)	15.0 (15.0, 48.6)	0.8	0.8
**Cortisol (ng/dL)**	11.3 (5.2, 28.7)	13.4 (5.8, 22.6)	0.2	0.1
**Cortisone (ng/dL)**	2.03 (0.97, 2.80)	1.97 (1.03, 2.73)	0.8	0.5
**18 Corticosterone (ng/dL)**	39.9 (7.8, 85.6)	39.9 (7.8, 85.6)	0.8	0.3
**DHEA (ng/dL)**	502.5 (165.7, 1217.0)	389.8 (67.5, 1093.8)	0.2	0.4
**DHEAS (μg/dL)**	171 (75, 366)	194 (36, 378)	0.7	0.1
**LH (IU/L)**	27.6 (10.8, 90.4)	10.3 (6.0, 21.9)	<0.001	0.002
**FSH (IU/L)**	10.7 (4.7, 16.9)	10.4 (6.6, 16.6)	0.8	0.1

1 p value corrected controlled for age and BMI, as appropriate.

2 Values reported as median (2.5, 97.5th %ile).

3 p value not corrected for BMI.

**Figure 1:**
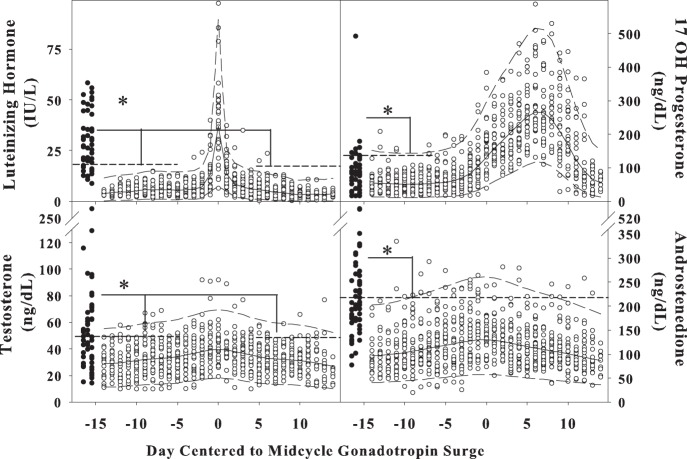
Hormones Across the Cycle in Controls and on a Single Day in Women with PCOS. A) LH, B) 17OH progesterone, C) testosterone and D) androstenedione levels in controls across the menstrual cycle (white circles) and women with PCOS on a day at least 10 days after the last menstrual cycle (black circles). Line plots are smoothly changed ranges of smooth model fittings using raw cycle day as the independent variable and transformed steroid as dependent variable. Solid black lines are back-transformed means and dashed lines are back-transformed mean±2SD. SDs are estimated using model residuals. The levels in women with PCOS are higher at all cycle phases indicated by the dotted lines and asterisks.

To examine data across the menstrual cycle in healthy women, data were transformed to the power of lambda and fitted against cycle day using smooth curve fitting (spline smooth) for the mean using raw cycle day as the sole independent variable and transformed steroid as the dependent variable (Figure 1 and Table S4 in in File S1). To plot the standard deviation, either a fixed standard deviation was estimated using the residuals about the regression mean or a polynomial regression model against cycle day was fitted to the standard deviations of the residuals [16]. The R quantile function (R2.11.1 (http://cran.r-project.org)) was used for smooth model fitting and to determine the 97.5% confidence limit for all measurements. The limit of quantification (LOQ) value was determined as the lower boundary of the assays. For results less than the LOQ, the LOQ was used for calculations.

## Results

Control subjects and women with PCOS had similar age and BMI (Table 1).

**Figure 2:**
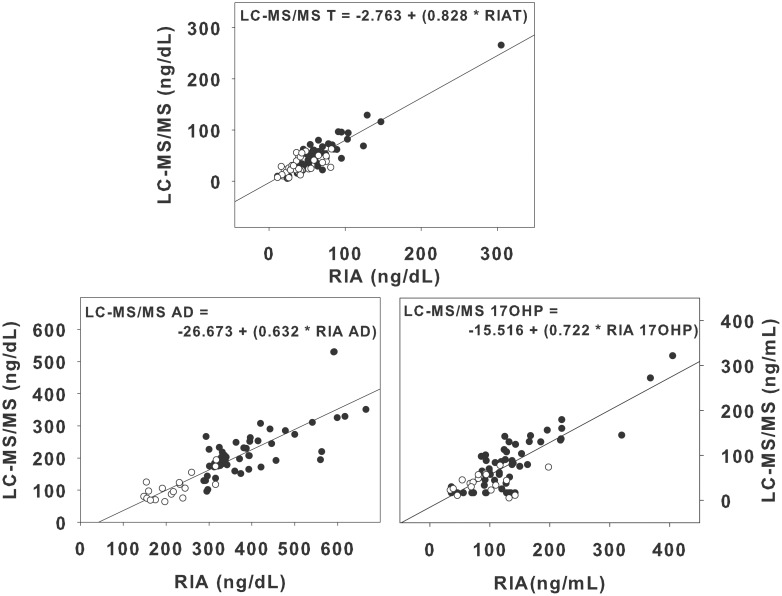
Hormone Levels Assayed Using LC-MS/MS and RIA. Testosterone (T), androstenedione (AD) and 17OH progesterone (17OHP) levels in individual subjects with PCOS (black circles) and controls (white circles) as measured in an RIA and LC-MS/MS. Linear regression is plotted for each hormone. Testosterone, androstenedione and 17-hydroxyprogesterone levels are consistently higher using the RIA.

**Figure 3:**
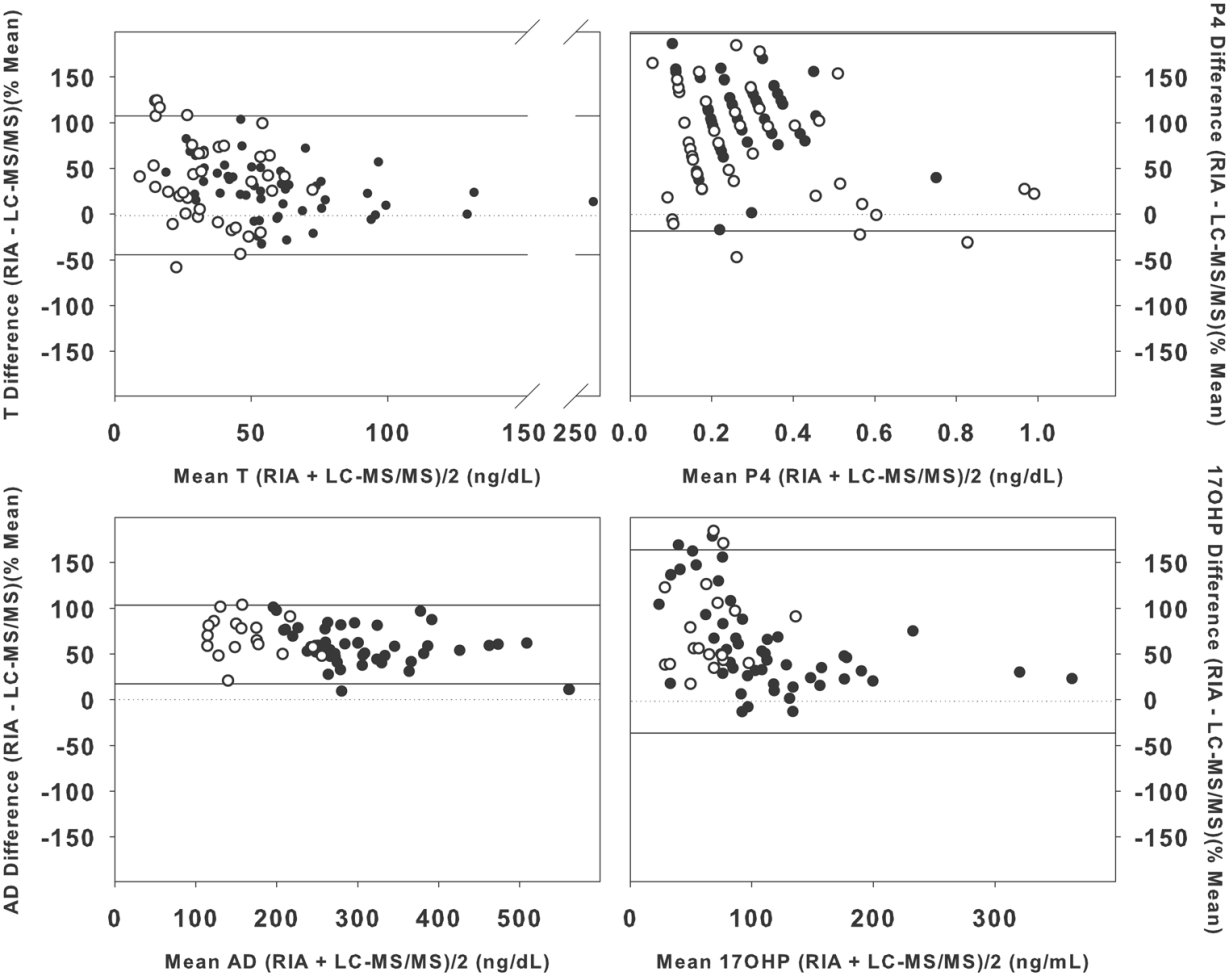
Percent difference in testosterone (T), androstenedione (AD) and 17-hydroxyprogesterone. (17OHP) levels measured in a RIA and LC-MS/MS measured in women with PCOS (black circles) and controls (white circles) as a function of mean levels measured in the two assays (Bland-Altman Plot). The solid lines indicate 2 standard deviations above and below the mean difference.

The slope of the fitted lines between the LC-MS/MS and RIA measurements were all less than 1, indicating that testosterone, androstenedione, and 17-hydroxyprogesterone levels were lower in the LC-MS/MS compared to the RIAs (Figure 2). The Bland-Altman plots demonstrated that the percent difference between the RIAs and the LC-MS/MS ranged from −58 to 186% (Figure 3). The average percent difference in levels between the RIAs and LC-MS/MS was lowest for testosterone (31±38%; mean±SD), and highest for progesterone measured in the follicular phase (89±54%), while androstenedione and 17-hydroxyprogesterone had similar average percent differences (60±22 and 63±50%, respectively). There was no difference in the percent difference in RIA and LC-MS/MS results in women with PCOS compared to controls for testosterone, androstenedione and 17-hydroxyprogesterone (all p>0.05). Progesterone levels were more variable in women with PCOS (106±41%) than in controls (71±60%; p<0.01).

Testosterone, androstenedione and 17-hydroxyprogesterone levels were higher in women with PCOS compared to follicular phase levels in controls, but other hormone levels were not different (Table 1 and Figure 1). LH levels were also higher in women with PCOS compared to controls, but there was no difference in FSH levels. As all hormone measurements were performed between 8 and 9 a.m., when ACTH is at its morning peak, ratios of the concentration of products-to-precursors were compared as a proxy for enzyme function in women with PCOS and controls. The concentration of total androgens and their precursors was greater for women with PCOS than controls (1607 (638, 3085) vs. 1143 (511, 4784) ng/dL; p = 0.03). The percent product was higher for 3β-hydroxysteroid dehydrogenase in women with PCOS compared to controls (Table 2). There was no difference in adrenal steroid concentrations, including DHEAS, deoxycorticosterone, corticosterone, 11 deoxycortisol and cortisol in women with PCOS compared to controls.

**Table 2:** Percent product-to-precursor concentrations for adrenal and ovarian enzymes in women with PCOS and controls. Data are expressed as % (2.5, 97.5% ile).

	PCOS	Controls	P Value
**3β-hydroxysteroid dehydrogenase** 1	22 (6, 92)	20 (4, 43)	0.008
**17α-hydroxylase**	861 (54, 6104)	953 (54, 10,345)	0.5
**17,20 lyase**	159 (25, 708)	161 (7, 551)	0.6
**17β-hydroxysteroid dehydrogenase**	22 (10, 52)	22 (9, 44)	0.4
**21 hydroxylase**	50 (22, 215)	86 (25, 278)	0.1
**11β-hydroxylase**	30708 (13229, 90147)	40419 (5514, 73264)	0.4

1 Results are expressed as % product to precursor.

## Discussion

The study demonstrates the development of a LC-MS/MS that can measure multiple adrenal and gonadal steroids simultaneously. The data demonstrate that LC-MS/MS testosterone, androstenedione, progesterone and 17-hydroxyprogesterone levels are lower than those measured using RIAs. Using LC-MS/MS to measure steroid levels from the adrenals and ovaries simultaneously, women with PCOS have higher testosterone, androstenedione and 17OH progesterone levels, but no difference in predominantly adrenal steroids. While not a measure of enzyme activity, the product-to-precursor ratios point to the possibility that 3β-hydroxysteroid dehydrogenase has increased activity in women with PCOS. These patterns of increased androgens measured simultaneously are consistent with an ovarian source of androgen production in women with PCOS, with direct testing needed.

Comparing RIAs and immunoassays with LC-MS/MS for four steroids, it was clear that the RIAs read consistently higher compared to the LC-MS/MS. The higher levels are likely related to interference by cross-reacting steroids and matrix effects [7], and these effects are decreased by extraction and chromatography preceding the RIA [9]. However, inaccurately higher testosterone levels in an RIA have been demonstrated previously, even with preceding extraction, as has the disparity between assays when compared across the normal female range [8], [9]. Progesterone levels in the follicular phase range demonstrated the greatest discrepancy between assays, which may be an important consideration if the rise in progesterone becomes an important factor to predict IVF success [17], [18]. Of note, testosterone levels using the RIA were associated with the lowest error, on average 31%, in the current study and similar to previous testosterone errors [9]. Further, there was no difference in the discrepancy between assays in women with PCOS and controls. Taken together, the relatively low percent difference between RIA and LC-MS/MS for testosterone measurements suggests that the RIA could remain an acceptable assay for studies involving women with PCOS.

In women with PCOS, 17OH progesterone, androstenedione and testosterone levels are elevated compared to those in control women. These data expand earlier findings comparing hormone levels in the follicular phase using a radioimmunoassay [19], [20]. The higher 17OH progesterone, androstenedione and testosterone levels could represent a contribution from the ovary or the adrenal gland. However, the current data in which DHEA, DHEAS and other predominantly adrenal steroids were not different between women with PCOS and control women support the ovary as the source of hyperandrogenism in PCOS [21], along with previous data [22], [23].

Further evidence for the ovarian source of androgens in PCOS comes from studies of isolated theca cells from women with PCOS and control women [3], [24]. Progesterone, 17OH progesterone, DHEA, androstenedione and testosterone levels were elevated in theca cells from women with PCOS compared to those from controls [2], [3]. In addition, side chain cleavage, 3β-hydroxysteroid dehydrogenase and 17α-hydroxylase/17,20 desmolase enzymes have increased activity in theca cells of women with PCOS [3], [25]. In the current data, higher 17OH progesterone, androstenedione and testosterone levels, but not progesterone or DHEA are consistent with the *in vitro* findings in theca cells. While product-to-precursor ratios do not measure enzyme activity, the greater androgen steroid concentration and androgen product-to-precursor ratio for 3β-hydroxysteroid dehydrogenase in women with PCOS compared to controls are also consistent with data from isolated theca cells. In contrast, the product-to-precursor ratio was not increased for adrenal-corticosteroid producing enzymes in women with PCOS. These data require direct testing of enzyme activity for confirmation.

One limitation of the current study is that steroids were measured in the absence of exogenous stimulation. However, the PCOS and control subjects did have their blood drawn in the morning, fasting, so that the levels reflect endogenous morning ACTH stimulation [5]. Previous studies demonstrate that urinary steroid metabolites produced by the 5α-reductase pathway [26] and the 11β-hydroxysteroid dehydrogenase pathway are increased in women with PCOS compared to controls in the early follicular phase, suggesting increased cortisol production and clearance [27]. The current study was unable to demonstrate differences in the cortisol and corticosterone levels, although steady state levels may not reflect differences if production and clearance are both upregulated in one of the groups. In addition, ratios were used to estimate enzyme activity rather than using direct biochemical measurements. Therefore, absolute conclusions regarding enzyme activity cannot be made.

The current study utilizes a new LC-MS/MS capable of measuring a large number of steroids from a small volume [28]. The assay may provide greater ability to distinguish ovarian hyperandrogenism in women with PCOS from other disorders in which adrenal steroidogenesis is compromised, such as in nonclassic congenital adrenal hyperplasia. The data confirm the excess testosterone, androstenedione and 17OH progesterone levels in women with PCOS and provide capability to examine adrenal and ovarian steroids by measuring all steroid levels in a single sample. The data confirm androgen excess in PCOS, with little difference in adrenal-predominant steroids. Future studies should explore enzyme activity directly in ovarian and adrenal tissue to determine the precise source of production.

## Supporting Information

File S1 **This includes Figure S1 and Tables S1 to S4.** Figure S1. Testosterone levels (n = 359) measured in the same radioimmunoassay on fresh serum (X axis) and on serum stored at −20° to −30°C for 14 years in a biobank (Y axis). The line of identity is indicated by the grey line and the linear regression of the measurements is indicated by the blue line. Table S1. Within Run Precision (Intra-Run Precision). Table S2. Total Precision (Inter-Run Precision). Table S3. Calibration Verification (Linearity). Table S4. Steroid concentrations across the menstrual cycle in control subjects.
(DOCX)
