# Supplementary material for: Simultaneous Measurement of Thirteen Steroid Hormones in Women with Polycystic Ovary Syndrome and Control Women Using Liquid Chromatography-Tandem Mass Spectrometry
Source: PLoS One. 2014 Apr 8;9(4):e93805. doi: 10.1371/journal.pone.0093805 (PMC3979722; doi:10.1371/journal.pone.0093805)
Supplement: File S1 — This includes Figure S1 and Tables S1 to S4. Figure S1. Testosterone levels (n = 359) measured in the same radioimmunoassay on fresh serum (X axis) and on serum stored at −20° to −30°C for 14 years in a biobank (Y axis). The line of identity is indicated by the grey line and the linear regression of the measurements is indicated by the blue line. Table S1. Within Run Precision (Intra-Run Precision). Table S2. Total Precision (Inter-Run Precision). Table S3. Calibration Verification (Linearity). Table S4. Steroid concentrations across the menstrual cycle in control subjects. (DOCX) [file pone.0093805.s001.docx]

Supplemental Table S1. Within Run Precision (Intra-Run Precision)

The Intra-Assay Variation is defined as the reproducibility of a sample within an assay and was generated from assaying 10 replicates from each the three QC pools.

The coefficient of variation (CV) for 10 replicates of a sample was used to determine if the reproducibility is acceptable (≤ 15% CV). Statistics performed on the results determined that the reproducibility (CV) for the three QC pools for each analyte is as follows:

| ***Analyte*** | Precision (% CV) | | |
| --- | --- | --- | --- |
|  | ***Level 1*** | ***Level 2*** | ***Level 3*** |
| 11-Deoxycortisol | 8.3 | 3.3 | 3.0 |
| 17-OH Progesterone | 4.9 | 3.4 | 4.5 |
| 17-OH Pregnenolone | 8.4 | 7.7 | 6.7 |
| 18-OH Corticosterone | 11.6 | 10.1 | 7.4 |
| Androstenedione | 6.5 | 4.4 | 4.8 |
| Corticosterone | 9.1 | 7.4 | 3.7 |
| Cortisol | 9.6 | 8.3 | 5.5 |
| Cortisone | 8.0 | 4.9 | 2.7 |
| DHEA | 13.2 | 9.9 | 9.1 |
| Deoxycorticosterone | 7.1 | 4.6 | 4.2 |
| Pregnenolone | 8.5 | 9.8 | 5.9 |
| Progesterone | 6.1 | 3.4 | 4.4 |
| Testosterone | 6.1 | 5.5 | 3.4 |

Supplemental Table S2. Total Precision (Inter-Run Precision)

The Inter-Assay Variation is defined as the reproducibility of a sample among assays and was generated from assaying 10 replicates from each of the three QC pools over 5 days or runs.

The coefficient of variation (CV) for multiple replicates (n=50) of a sample was used to determine if the reproducibility is acceptable (≤ 15% CV). Statistics performed on the results determined that the reproducibility (CV) for the three QC pools for each analyte are as follows:

| ***Analyte*** | Precision (% CV) | | |
| --- | --- | --- | --- |
|  | ***Level 1*** | ***Level 2*** | ***Level 3*** |
| 11-Deoxycortisol | 9.6 | 7.8 | 7.3 |
| 17-OH Progesterone | 11.2 | 6.3 | 6.1 |
| 17-OH Pregnenolone | 10.3 | 11.7 | 13.9 |
| 18-OH Corticosterone | 13.2 | 9.3 | 9.3 |
| Androstenedione | 9.1 | 6.5 | 6.1 |
| Corticosterone | 12.4 | 9.1 | 5.5 |
| Cortisol | 12.2 | 11.5 | 8.7 |
| Cortisone | 10.8 | 7.7 | 7.5 |
| DHEA | 12.9 | 11.3 | 9.0 |
| Deoxycorticosterone | 12.6 | 7.3 | 6.3 |
| Pregnenolone | 10.7 | 13.1 | 7.8 |
| Progesterone | 8.6 | 8.6 | 7.4 |
| Testosterone | 8.5 | 10.6 | 4.6 |

Supplemental Table S3. Calibration Verification (Linearity)

| *Analyte* | *Date* | *Calibration Range* | *Math Model & Weighting* | *Corr.*  *Coeff.* |
| --- | --- | --- | --- | --- |
| 11-Deoxycortisol | 08/04/10 | 25 – 10,000 ng/dL | Linear, 1/x | 0.9994 |
| 17-Hydroxyprogesterone | 08/05/10 | 25 – 10,000 ng/dL | Linear, 1/y | 0.9990 |
| 17-Hydroxypregnenolone | 08/04/10 | 50 – 10,000 ng/dL | Linear, 1/x or Equal | 0.9975 |
| 18-Hydroxycorticosterone | 07/06/10 | 50 – 10,000 ng/dL | Linear, 1/x or 1/y | 0.9986 |
| Androstenedione | 07/05/10 | 25 – 10,000 ng/dL | Linear, 1/x or 1/y | 0.9989 |
| Corticosterone | 08/03/10 | 25 – 10,000 ng/dL | Linear, 1/x or 1/y | 0.9997 |
| Cortisol | 07/06/10 | 0.05 – 10.0 ug/dL | Linear, 1/x or 1/y | 0.9985 |
| Cortisone | 08/03/10 | 0.025 – 10.0 ug/dL | Linear or Quad, 1/y | 0.9996 |
| Dehydroepiandrosterone | 07/09/10 | 50 – 10,000 ng/dL | Linear, 1/x | 0.9993 |
| Deoxycorticosterone | 08/03/10 | 25 – 10,000 ng/dL | Linear, 1/x or 1/y | 0.9991 |
| Pregnenolone | 07/07/10 | 50 – 10,000 ng/dL | Linear, 1/x or 1/y | 0.9994 |
| Progesterone | 08/05/10 | 0.25 – 100.0 ng/mL | Linear or Quad, 1/x or1/y | 0.9992 |
| Testosterone | 08/11/10 | 25 – 10,000 ng/dL | Linear, 1/y or Equal | 0.9993 |

Supplemental Table S4. Steroid concentrations across the menstrual cycle in control subjects.

| Hormone | LOQ^1^ | EFP^2^ | MFP | LFP | MGS | ELP | MLP | LLP | P value^3^ |
| --- | --- | --- | --- | --- | --- | --- | --- | --- | --- |
| Progesterone (ng/mL) | 0.08 | 0.18  (0.08, 2.85)^a 4,5^ | 0.12  (0.08, 0.25) ^a^ | 0.14  (0.08, 0.87) ^a^ | 0.70  (0.10, 1.48) ^a^ | 4.88  (1.23, 7.76) ^b^ | 15.04  (6.68, 22.20) ^c^ | 6.61  (3.31, 19.09) ^d^ | <0.001 |
| 17 OH Progesterone (ng/dL) | 17 | 52.0  (20.3, 92.8) ^a^ | 58.8 (23.2, 102.2) ^a^ | 68.6 (33.8, 140.4) ^a^ | 177.0 (66.8, 348.8) ^b,d^ | 183.3 (104.8, 287.3) ^b^ | 260.8 (139.2, 431.1) ^c^ | 132.6 (86.7, 301.3) ^d^ | <0.001 |
| Androstenedione (ng/dL) | 9 | 88.2  (57.5, 181.7)^a^ | 112.8 (50.5, 212.9) ^b,c^ | 123.9 (78.8, 216.0) ^c,d^ | 136.0 (72.5, 230.0) ^d^ | 122.9 (86.8, 183.6) ^b,d^ | 109.4 (73.4, 184.1) ^b^ | 97.6 (67.8, 175.3) ^a,b^ | <0.001 |
| Testosterone (ng/dL) | 10 | 26.3  (16.2, 47.4)^a^ | 33.1 (14.4, 52.5) ^a^ | 39.1 (21.5, 53.6) ^b,d^ | 42.0 (24.2, 66.2) ^b-d^ | 38.5 (24.7, 54.3) ^b,c^ | 34.3 (21.1, 47.3) ^a,d^ | 30.1 (19.8, 53.6) ^a^ | <0.001 |
| Estradiol (pg/mL) | 2 | 32.67 (7.42, 94.7)^a^ | 40.5 (6.3, 87.2)^a,b^ | 112.8 (26.9, 181.7)^c^ | 162.0 (44.4, 373.4)^d^ | 70.3 (18.3, 117.9)^b,c^ | 108.5 (45.6, 219.2)^d^ | 76.4 (30.5, 178.7)^a,b^ | <0.001 |
| Pregnenolone (ng/dL) | 35 | 67.4  (35, 305.4) | 72.5 (35, 285.1) | 69.7 (35, 236.1) | 58.0 (35, 256.8) | 63.8 (35.5, 404.9) | 62.9 (35, 223.5) | 59.2 (35, 535.2) | 0.9 |
| 17 OH Pregnenolone (ng/dL) | 33 | 182.2 (33, 365.2) | 192.4 (38.4, 377.9) | 194.2 (63.7, 551.3) | 124.0 (33, 582.8) | 186.4 (38.7, 459.7) | 159.1(69.9, 470.1) | 143.4 (45.0, 458.1) | 0.4 |
| Deoxycorticosterone (ng/dL) | 16 | 16 (16, 22) | 16 (16, 18) | 16 (16, 19) | 16 (16, 23) | 16 (16, 20) | 16 (16, 19) | 16 (16, 202) | 0.3 |
| Corticosterone (ng/dL) | 17 | 274.9 (81.1, 778.7) | 292.5 (90.1, 942.0) | 294.4 (82.0, 953.4) | 193.0 (38.0, 1422.4) | 297.8 (65.9, 1009.6) | 351.5 (94.9, 791.2) | 232.1 (111.0, 972.4) | 0.2 |
| 11 Deoxycortisol (ng/dL) | 15 | 19.1 (15, 55.1) | 22.4 (15, 45.7) | 21.2 (15, 59.6) | 15.0 (15, 69.2) | 17.2 (15, 72.9) | 20.2 (15, 39.9) | 17.4 (15, 48.9) | 0.1 |
| Cortisol (mcg/dL) | 0.025 | 12.4 (7.5, 19.7) | 13.2 (6.3, 19.7) | 11.1 (8.0, 20.2) | 12.6 (2.8, 24.6) | 11.0 (4.7, 21.8) | 11.5 (3.75, 20.1) | 10.6 (6.1, 20.7) | 0.1 |
| Cortisone (mcg/dL) | 0.015 | 2.4 (1.5, 3.1) | 2.3 (1.2, 3.3) | 2.4 (1.4, 3.5) | 2.4 (0.8, 3.3) | 2.2 (1.0, 3.0) | 2.3 (1.0, 3.1) | 2.1 (1.2, 3.1) | 0.02 |
| Dehydroepiandrosterone (ng/dL) | 23 | 634.4 (297.1, 1060.3) | 716.1 (385.4, 1143.4) | 735.3 (345.2, 2029.5) | 616.0 (345.2, 2029.5) | 714.7 (445.3, 1157.6) | 769.1 (414.4, 1294.5) | 661.8 (328.8, 1239.3) | 0.6 |

^1^ LOQ-limit of quantification

^2^ EFP=early follicular phase; MFP=midfollicular phase; LFP=late follicular phase; ELP=early follicular phase; MLP=mid follicular phase; LLP=late follicular phase

^3^Repeated measures ANOVA p value

^4^Data are expressed as median (2.5, 97.5 percentiles)

^5^Different letters indicate significant differences

Supplemental Figure S1. Testosterone levels (n=359) measured in the same radioimmunoassay on fresh serum (X axis) and on serum stored at -20° to -30° C for 14 years in a biobank (Y axis). The line of identity is indicated by the grey line and the linear regression of the measurements is indicated by the blue line.
